# The effect of psychotic proneness and psychopathy on theory of mind

**DOI:** 10.1002/pchj.689

**Published:** 2023-10-20

**Authors:** Begüm Atakan, Elif Yildirim

**Affiliations:** ^1^ Department of Psychology Isik University Istanbul Turkey

**Keywords:** anti‐social behavior, psychopathy, psychosis, social cognition, theory of mind

## Abstract

This study aimed to investigate the interaction between positive psychotic experiences and psychopathic traits on the theory of mind in a non‐clinical sample. The results showed that distinct constructs of psychopathy can lead to distinct theory of mind profiles when interacting with psychotic proneness.

It is known that theory of mind (ToM) is impaired in psychotic disorders and psychopathy. However, the impact of the co‐occurrence of psychotic symptoms and psychopathic traits on ToM remains a subject of controversy. Some reports showed that the co‐occurrence of these symptoms caused double‐dose impairment in judging others' emotions based on facial expression, which exceeded the impairment seen in either symptom alone (Fullam & Dolan, 2006). Other reports have suggested that this co‐occurrence does not always result in further decline but, in some cases, can even preserve or enhance ToM (Gillespie et al., 2017).

The conflicting findings may be attributed to the methods used to assess psychopathic traits. Many studies have focused on psychopathy as a single construct or have only measured primary psychopathic traits. However, evidence suggests that different psychopathic constructs have distinct effects on ToM performance (Song et al., 2023). Primary psychopathic traits, such as shallow affect, emotional detachment, and difficulties in forming deep relationships, have been suggested to be associated with intact affective ToM, whereas secondary psychopathic traits, which encompass features related to an antisocial lifestyle, such as susceptibility to boredom, impulsivity, and emotional dysregulation, have been linked to lower abilities in both cognitive and affective ToM (Sharp & Vanwoerden, 2014). In this context, the present study aimed to examine the interaction between both primary and secondary psychopathic traits and positive psychotic experiences on affective ToM in a non‐clinical sample. By focusing on a non‐clinical sample, this study sought to avoid confounding effects related to clinical factors such as disease‐specific symptoms and medication treatments.

The sample comprised 593 adults, including 378 (64%) females and 215 (36%) males. The age range of the participants was 18 to 65 years, with a mean age of 29.45 years (*SD* = 11.49). Participants were recruited through advertisements on social media platforms, and the data were collected via an online platform. To assess ToM performance, we used the Reading the Mind in the Eyes Test (RMET) (Baron‐Cohen et al., 2001). The original RMET consisted of 36 photographs, but four photographs were removed in the Turkish version of the RMET. Levenson Self‐Report Psychopathy Scale (LSRP) (Levenson et al., 1995), which is a 26‐item self‐report measure, was used to assess primary psychopathy (P‐LSRP) and secondary psychopathy (S‐ LSRP). The Community Assessment of Psychic Experience (CAPE), which includes 42 items, is used to evaluate the presence of psychosis symptomatology (Stefanis et al., 2002). This study used the CAPE's 20‐item Positive subscale (CAPEp) to measure psychotic proneness.

Two multiple regression analyses were conducted to test the effects of the interaction of LSRP and CAPEp on the RMET score. Control variables (age and gender) were included in the first step of each regression, whereas LSRP, CAPE, and LSRP × CAPE (interaction) were added to the second step. To avoid high multicollinearity with the interaction term, the variables were mean‐centered and an interaction term between these variables was computed. The method, which entails examining the effect of one RMET predictor at another predictor's mean, as well as 1 standard deviation above and 1 below the mean, was applied to probe significant interactions.

The results of the regression analysis that examined the interaction between P‐LSRP and CAPEp for predicting RMET performance showed that the overall regression model was significant (*F*(5, 587) = 6.93, *p* < .001), explaining 5% of the variance. The variables added to the second step contributed to a significant change in variance (*F*_change_ (3587) = 6.52, *p* < .001). Parameter estimates indicated a relation of RMET with P‐LSRP (*β* (*SE*) = −.08 (.03), *t* = −2.96, *p* = .003), but there is no significant interaction of CAPEp with P‐LSRP (*p* > .05). Second analysis conducted to investigate the interaction between S‐LSRP and CAPEp revealed that the overall regression model was significant (*F*(5, 587) = 9.81, *p* < .001), explaining 8% of the variance. The variables added to the second step were found to have contributed to a change in variance (*F*_change_ (3587) = 11.21, *p* < .001). According to parameter estimates, RMET was significantly associated with CAPEp (*β* (*SE*) = −.78 (.24), *t* = −3.29, *p* = .001) and S‐LSRP (*β* (*SE*) = −.22 (.04), *t* = −4.84, *p* < .001); however, an interaction of CAPEp with S‐LSRP accounted for this effect (*β* (*SE*) = .03 (.01), *t* = 3.05, *p* = .002).

Figure 1A displays the significant relationships between more positive psychotic experience and worse ToM performance with low (*β* (*SE*) = −.24 (.07), *t* = −3.43, *p* < .001, CI95 = −.38 to −.10) and moderate S‐LRSP (*β* (*SE*) = −.12 (.05), *t* = −2.72, *p* = .007, CI95 = −.21 to −.03). Similarly, Figure 1B shows that the relationships between S‐LRSP and RMET were significant when CAPEp was low (*β* (*SE*) = −.22 (.04), *t* = −4.84, *p* < .001, CI95 = −.30 to −.13) and moderate (*β* (*SE*) = −.12 (.03), *t* = −3.64, *p* < .001, CI95 = −.19 to −.06).

**FIGURE 1:**
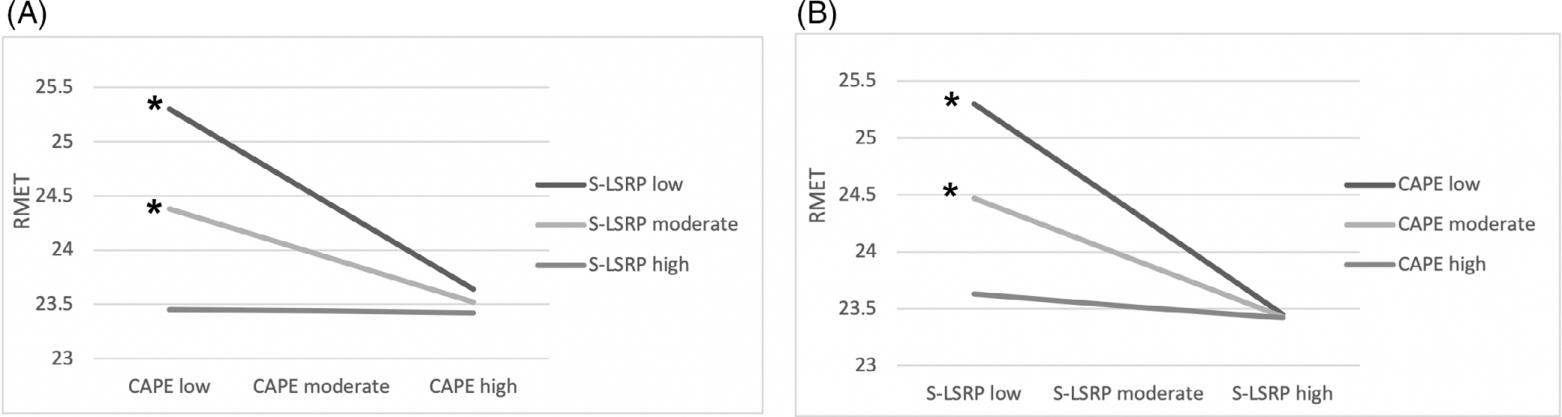
(A) Visualization of the interaction between positive psychotic experiences (Community Assessment of Psychic Experience [CAPE]) and affective theory of mind (Reading the Mind in the Eyes Test [RMET]) in plots of simple regression lines for the participants with low (*M* = 19.54), moderate (*M* = 23.83), and high (*M* = 28.12) secondary psychopathy (Levenson Self‐Report Psychopathy Scale [S‐LSRP]). (B) Visualization of the relationship between RMET and S‐LSRP when CAPEp scores are low (*M* = .00), moderate (*M* = 3.44), and high (*M* = 6.97). * indicates a significant relationship (*p* < .05).

Consistent with previous studies (Gillespie et al., 2017), our findings revealed that higher levels of positive psychotic experiences, as well as primary and secondary psychopathic traits, were associated with impaired ToM performance. Although primary psychopathic traits did not affect the association between positive psychotic experiences and ToM, our findings provided evidence of deleterious effects of the co‐occurrence of psychotic proneness and secondary psychopathic traits on ToM (Fullam & Dolan, 2006). These findings could be explained by distinct neurocognitive mechanisms through which primary and secondary psychopathy negatively impact social cognition.

Previous research has indicated that while primary psychopathic traits are associated with reduced responsiveness to others' distress, secondary psychopathic traits are linked to heightened responsiveness to others' emotional states (Sethi et al., 2018). This heightened responsivity related to secondary psychopathy may contribute to the exacerbation of ToM impairments caused by positive psychotic symptoms. However, it appears that this effect diminishes when secondary psychopathic traits reach a high level. The presence of elevated psychopathic traits may limit further deterioration in affective ToM.

In conclusion, the findings of this study suggest that different constructs of psychopathy create distinct ToM profiles when interacting with psychotic proneness. Although these findings need to be replicated within clinical populations to make any conclusion about the co‐occurrence of psychosis and psychopathy, they have potential implications for clinical assessments. Our findings suggest that using the psychopathy total score may provide a limited understanding of the relationship between psychopathy and ToM, as psychopathy is a multidimensional construct (Song et al., 2023).

## CONFLICT OF INTEREST STATEMENT

The authors have no relevant financial or non‐financial interests to disclose.

## ETHICS STATEMENT

The questionnaire and methodology for this study were approved by the Ethics Committee of Isik University (E‐59426783‐900‐6765). Informed consent (online) was obtained from all individual participants included in the study.
